# How to Measure Intraocular Pressure: An Updated Review of Various Tonometers

**DOI:** 10.3390/jcm10173860

**Published:** 2021-08-27

**Authors:** Paolo Brusini, Maria Letizia Salvetat, Marco Zeppieri

**Affiliations:** 1 Department of Ophthalmology, Policlinico “Città di Udine”, 33100 Udine, Italy; brusini@libero.it; 2 Department of Ophthalmology, Azienda Sanitaria Friuli Occidentale, 33170 Pordenone, Italy; mlsalvetat@hotmail.it; 3 Department of Ophthalmology, University Hospital of Udine, 33100 Udine, Italy

**Keywords:** intraocular pressure (IOP), tonometry, Goldmann applanation tonometer (GAT), central corneal thickness (CCT), ocular hypertension, glaucoma

## Abstract

Intraocular pressure (IOP) is an important measurement that needs to be taken during ophthalmic examinations, especially in ocular hypertension subjects, glaucoma patients and in patients with risk factors for developing glaucoma. The gold standard technique in measuring IOP is still Goldmann applanation tonometry (GAT); however, this procedure requires local anesthetics, can be difficult in patients with scarce compliance, surgical patients and children, and is influenced by several corneal parameters. Numerous tonometers have been proposed in the past to address the problems related to GAT. The authors review the various devices currently in use for the measurement of intraocular pressure (IOP), highlighting the main advantages and limits of the various tools. The continuous monitoring of IOP, which is still under evaluation, will be an important step for a more complete and reliable management of patients affected by glaucoma.

## 1. Introduction

Intraocular pressure (IOP) is an important measurement, which should be taken in every patient over the age of 40 that undergoes a complete ophthalmic examination and in all patients with ocular hypertension (OHT) or with risk factors for developing primary open-angle glaucoma (POAG) (i.e., family history, myopia, increased cup-to-disc ratio, etc.). IOP measurement is obviously a fundamental tool in subjects with diagnosed ocular hypertension or glaucoma. Even if the IOP measurement in vivo is only an estimate of the true IOP (which is only possible with invasive manometry), this value, rightly or wrongly, is often taken as an indicator of the efficacy of any treatment for glaucoma and to assess glaucoma severity and progression in patient management. It is thus of great importance to acquire accurate and precise IOP measurements in clinical practice.

Numerous instruments, called tonometers, have been proposed since the 19th century to obtain IOP measurements [1,2,3]. Based on the operating principle, these instruments can be differentiated into two main groups: (1) indentation tonometers; (2) applanation tonometers.

## 2. Indentation Tonometry

**Figure 1:**
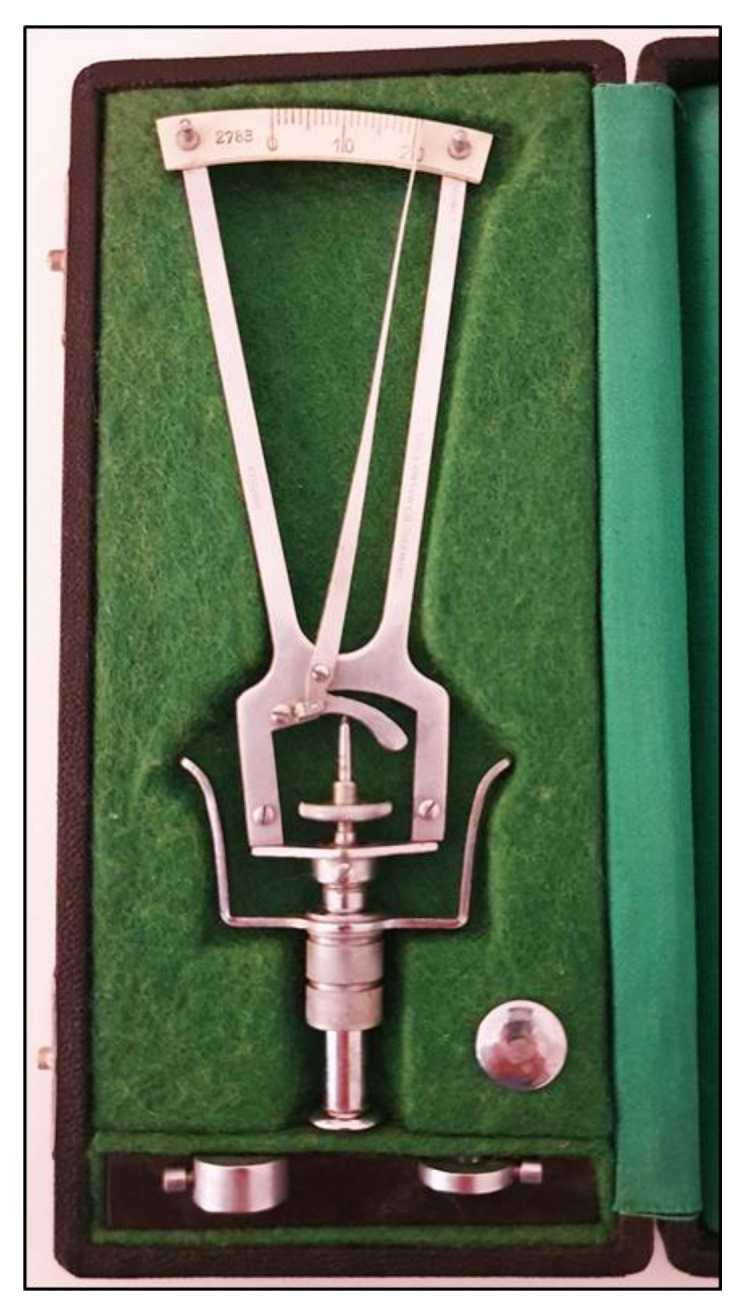
Schiøtz tonometer with different weights.

The prototype of the indentation tonometers is the Schiøtz tonometer that was introduced many years ago [4] and is no longer currently used (Figure 1).

Using this instrument, the cornea is indented by a plunger loaded with different weights. The IOP is based on the depth of indentation. The values are shown on a scale ranging from 0 to 20 units, in which the protrusion of the plunger of 0.05 mm represents each unit of measurement. The value indicated on the handle needs to be converted in mmHg using a conversion scale. The coefficient of ocular rigidity, which can differ amongst eyes, should be taken into consideration to obtain corrected measurements of IOP. The Schiøtz tonometer is a simple and relatively inexpensive instrument. It is still sometimes used in developing countries [5,6] and in children under general anesthesia [7]. This tonometer, however, is subject to several sources of error, which include improper positioning on the eye, defective or dirty instruments, high variability in comparison with other devices and measurements influenced by individual ocular rigidity [8]. Moreover, patients must be in a supine position when taking measurements with this tonometer.

## 3. Applanation Tonometry

Applanation tonometers are currently considered the most reliable instruments for an accurate IOP measurement. Such tonometers use the Imbert–Fick law: P = F/S, in which P is pressure, S represents the surface of the flattened area, and F is the force needed to flatten a fixed corneal area. Apart from the tonometer by Maklakoff and several other instruments that are no longer currently in use, in which the force is provided by the weight of the tonometer itself, applanation tonometry is based on the area of flattened cornea that is calculated and converted in mmHg [2]. In almost all instruments of this type, the F value is varied to get the proper corneal applanation for a predetermined area. The Goldmann applanation tonometer (GAT) was first invented in 1948 by Hans Goldmann [9] and is still considered the gold standard to date. The tonometer needs to be positioned on a slit lamp.

**Figure 2:**
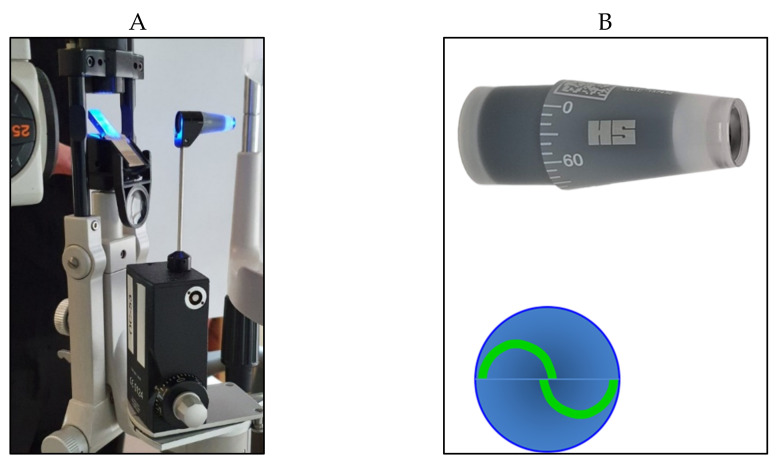
Goldmann applanation tonometer positioned on the slit lamp (**A**) with its cone prism (**B**) (on the top right); the two arcs appear correctly aligned (**B**) (on the bottom right).

A truncated cone, with a 7.35 mm^2^ surface area and a dimeter of 3.06 mm, illuminated by a blue light, is pushed on the center of the anaesthetized cornea. A doubling prism embedded in the cone divides the circular meniscus on the surface of the flattened cornea e into two arcs, which need to be aligned in order to obtain a precise and standardized applanation (Figure 2).

**Figure 3:**
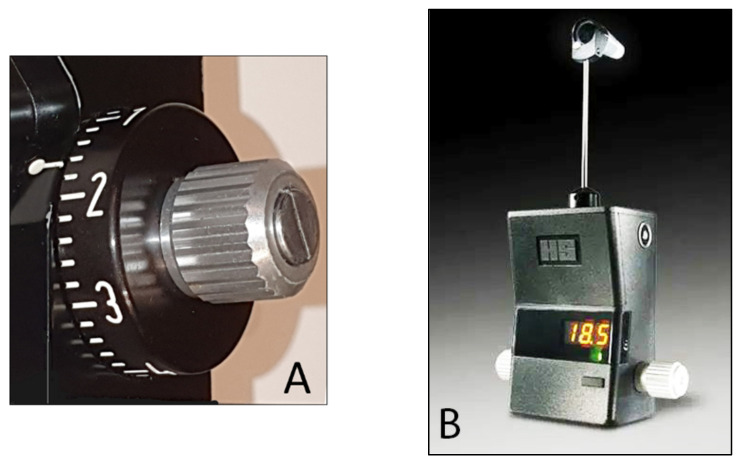
(**A**) Scale with IOP values in the Goldmann tonometer; (**B**) digital Goldmann tonometer (posterior view).

The force used needed to flatten the corresponding surface of the cornea is directly proportional to the IOP, expressed in mmHg that can be directly read in the scale of the measuring drum or in the posterior window for the digital version (Figure 3A,B).

Contrary to what Hans Goldmann believed, corneal thickness may show a significant effect on IOP measurements. Thin corneas can give rise to an underestimation of the IOP and vice versa. Several authors have tried to address this problem by proposing a number of correction formulae [10,11,12,13,14]; however, none have been shown to be of widespread use. Several corneal biomechanical properties, which are not all completely known, may be involved, thus rendering the proposed correction factors misleading and limiting their clinical use [15,16,17]. Studies have reported that a thin cornea can be a factor of risk for developing glaucoma [18], in addition to the underestimated IOP with GAT. 

GAT it is still the tonometer most commonly used in clinics, thanks to the ease of use, accuracy, reproducibility and affordability. There are, however, several drawbacks that should be kept in mind, as recently reported by Gazzard et al. [19]. GAT is affected by parameters of the cornea, which include central corneal thickness when this is far from the average (540 microns) [14,15], in addition to corneal curvature, axial length, hysteresis, etc. Moreover, GAT measurements are subjective and can depend on the physician experience. Studies have reported that even for the same physician, clinically significant differences can be found with a 95% repeatability coefficient of ±2 mmHg [20]. Other possible errors and drawbacks are due to the tear film with too little or too much fluorescein or an irregular or scarred cornea. GAT needs to be positioned on a slit lamp, and the subject must be in an upright position [21]. It is also important to remember that topic anesthesia is needed and that GAT should be periodically calibrated to provide good precision [22].

**Figure 4:**
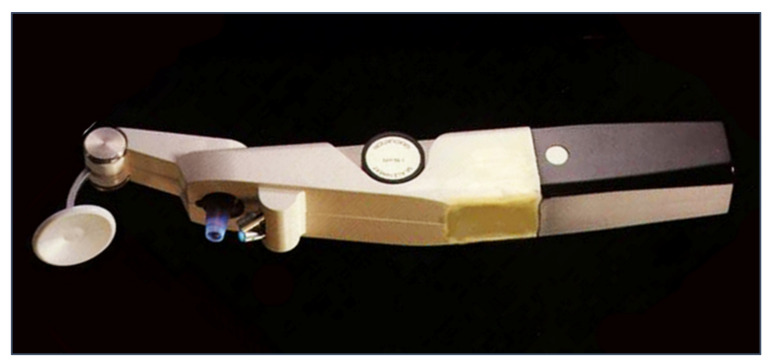
Handheld Perkins tonometer.

Portable handheld versions, such as the Perkins and Draeger tonometers (Figure 4), allow for measurements of IOP to also be taken supine and can be particularly useful in bedridden subjects and patients under general anesthesia. 

**Figure 5:**
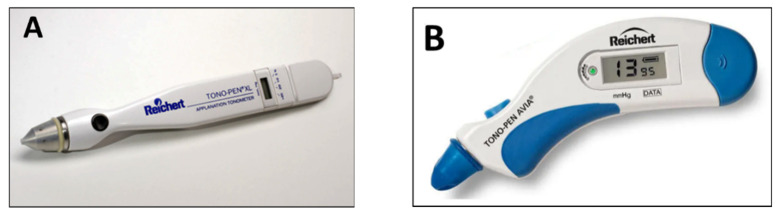
(**A**) Tono-Pen; (**B**) Tono-Pen Avia.

Other applanation instruments that use the same principle have been introduced several years ago. The Tono-Pen and the more recent Tono-Pen Avia (Reichert Ophthalmic Instruments, Depew, NY, USA) are portable lightweight battery-powered devices, which use the principles of applanation and indentation (Figure 5A,B). The reliability of each measurement is reported on a small display based on the standard deviation of the average of 10 readings. A disposable latex cap is used for each patient, which helps to reduces the risk of infection between patients.

Numerous studies have reported the usefulness of these devices in clinics in comparison with GAT [23,24,25,26], but the repeatability coefficients for intra-session repeated measurement have been shown to be quite high (±4.3 mmHg) [27]. Clinical studies have shown that Tono-Pen can be significantly affected by CCT [28]. This tonometry, however, seems to provide better accuracy in edematous corneas in comparison with GAT and dynamic contour tonometry [29]. It is important to note that different tonometers cannot be used interchangeably [30,31].

The advantages of Tono-Pen include portability and instrumentation that does not require a slit lamp or electricity. IOP readings can be measured in both supine and upright positions. Topo-Pen can be especially useful in patients with eye scarring or irregular corneas, and in children and bedridden subjects.

Studies have shown the clinical limits of this instrument. Tono-pen was found to consistently underestimate IOP, with a significant error for IOP values >30 mmHg [32]. Several concerns still remain regarding the reproducibility of measurements when used in a routine clinical setting, considering that significant variations from Goldmann readings may occur in some patients.

## 4. Non-Contact Tonometry (Air-Puff Tonometry)

**Figure 6:**
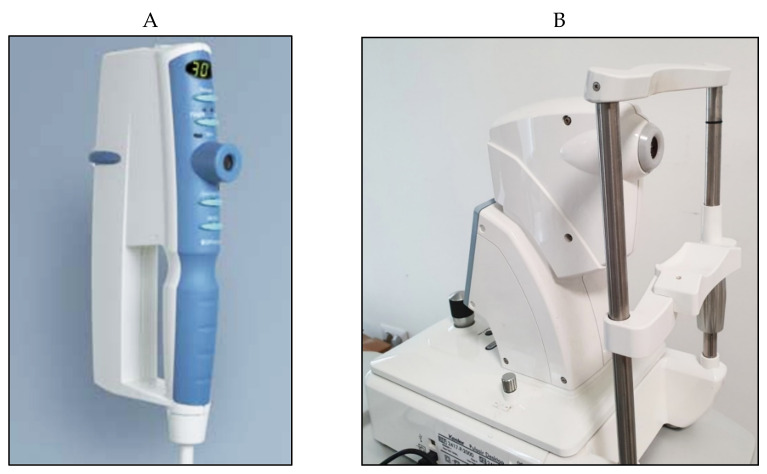
Pulsair EasyEye handheld (**A**) and Pulsair desktop (**B**) non-contact tonometers.

Non-contact tonometry (NCT) was first designed by Zeiss and developed by Grolman in 1972 [33]. Several models have been proposed in the past few decades that use a pulse of air to flatten the cornea without the need for touching the eye (Figure 6); such models, therefore, do not require anesthesia or fluorescein drops. In the Pulsair tonometer, a light beam is used in combination with a sensor that stops the production of air and measures the force used at the moment of corneal flattening.

Numerous studies have examined the differences in IOP measured with various types of NCT instruments and other non-conventional tonometers compared to GAT [34,35]. Demirci et al. showed that IOP measurements with NCT were significantly higher than those obtained with both GAT and rebound tonometry, with significant differences (*p* < 0.001) in all age groups [36]. A recent study confirmed that NCT tends to overestimate IOP GAT measurements in patients with IOP > 16 mmHg, which was more evident when IOP > 20 mmHg [37], showing a decrease in accuracy at higher values.

Early studies in 1989 based on the comparisons with GAT, showed that up to 70% of NCT measurements fell within ± 3 mmHg of readings taken with GAT. When using a screening criteria of IOP > 21 mmHg, NCT showed a sensitivity of 85% and a specificity of 95% [38].

Similar to GAT and other instruments, NCT is influenced by corneal parameters. Kyei et al. showed a significant association between CCT and NCT, which is greater than that reported with GAT [39]. These finding were confirmed in a recent study, which concluded that GAT measurements are not equivalent and cannot be interchanged with those obtained by NCT [40].

The pros of NCT are mostly based on the ease of use, non-contact nature and portability of several devices. Measurements can be taken by non-medical staff and patient compliance is relatively good in most casesNCT does not require slit-lamp positioning; thus, it is easily used in cases with elderly individuals, children, disabled patients and patients with limited collaboration. NCT can be considered for patients that may not tolerate topical anesthetics, patients with limited collaboration or those at greater risk of infection.

The disadvantages of NCT include the fact that NCT is less accurate when IOP > 20 mmHg. Studies have shown that NCT results depend on the instrument brand, unit and model of the device used [41]. Comparison studies between three NTC devices showed that, when taking GAT as the gold standard and aiming to detect IOP > 21 mmHg, sensitivities greatly differed from 40%, 48% and 80%, which showed that NCT readings are device dependent and that devices require regular calibration [38]. Although NCT offers a non-contact mode of measuring that limits the risk of infection due to contaminated drops or Goldmann prisms, the risk of air-borne infection could be greater considering the air-puff nature, which should be considered in the midst of the recent COVID era [42,43,44,45].

NCT could be helpful in a day-to-day clinical setting that involves dealing mostly with normal patients undergoing routine checkups. This type of tonometry can be ideal as a screening tool, which can easily be performed by non-medical staff. Although studies have shown that NCT tends to overestimate GAT measurements, NCT can prove to be useful for post-operative patients with lid edema, limited collaboration, ocular pain, discomfort and increased tear film meniscus size, which are all factors that influence proper GAT measurements. NCT can be a useful screening tool, but should never replace or be interchanged with GAT, especially in the management of patients with risk factors, ocular hypertension, suspect patients and glaucoma.

Other types of non-contact tonometers, with new interesting features, have recently been introduced. In addition to the traditional tonometers, these devices show IOP values that take CCT and corneal biomechanics into account, claiming to provide more accurate IOP measurements [19,46].

**Figure 7:**
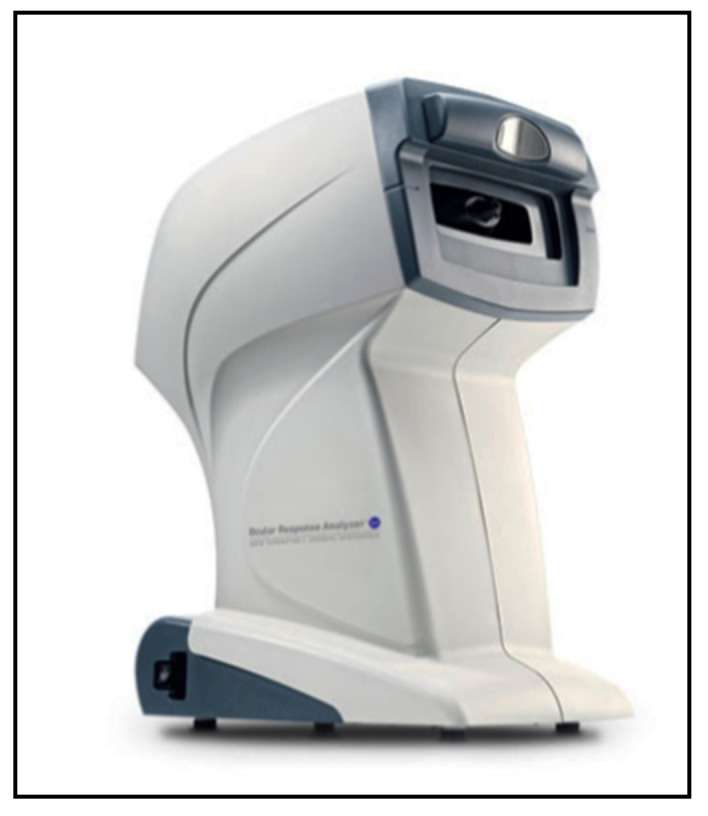
Ocular Response Analyzer (ORA) mod.G3.

The Ocular Response Analyzer (Reichert Technologies, Depew, NY, USA) or ORA, developed in 2005 by Luce et al. [46], is a non-contact air-puff tonometer that provides an optical electrical device to measure the deformation of the cornea caused by the impact of the air (Figure 7).

The force of the air makes the cornea move in an inward fashion in a first applanation state, causing it to take on a slight concave shape, to then move outward in a further applanation state, and finally to take on a normal configuration state. The electro-optical applanation detection system registers the curvature of the cornea in a diameter of 3 mm in the center for 20 msec. The two inward and outward applanation events, which are delayed by the viscoelastic corneal damping, allow for the calculations of two different IOP values based on the applanation principle. The instruments provide an average of these two pressure measurements and supply the so-called Goldmann-correlated IOP value (IOPg); the corneal hysteresis (CH) parameter is based on the difference between these two measurements of pressure. The instrument also provides a corneal-compensated IOP (IOPcc), which is based on the biomechanical properties of the cornea (elasticity and viscosity), to compensate for the measured IOP values [46,47].

There are several pros and cons to this device. The ORA is a relatively expensive device, but it is easy to handle, and topical anesthesia and fluorescein are not needed. In comparison with GAT, ORA has been demonstrated to significantly overestimate the IOP values, especially at high IOP levels [47,48]. Several authors have demonstrated that the IOPcc is less affected by corneal properties [48,49,50,51] and may better reflect the true IOP after refractive surgery of the cornea when compared to GAT IOP values [52].

Recent studies have shown that the ORA IOPcc values were superior to the GAT IOP measurements in predicting rates of glaucoma progression [53,54]. The CH parameter has been associated with other parameters of damage due to glaucoma, such as high cup-to-disc ratio and defects in the visual field. Several studies have shown a low CH value to be an independent predictor of functional damage occurrence or progression in the visual field in patients affected by ocular hypertension or glaucoma [55,56,57,58]. Moreover, the CH value can help in detecting patients with pathologies of the cornea such as keratoconus [59]. It may also be a useful parameter for patients at risk of developing corneal ectasia after refractive LASIK surgery [60].

**Figure 8:**
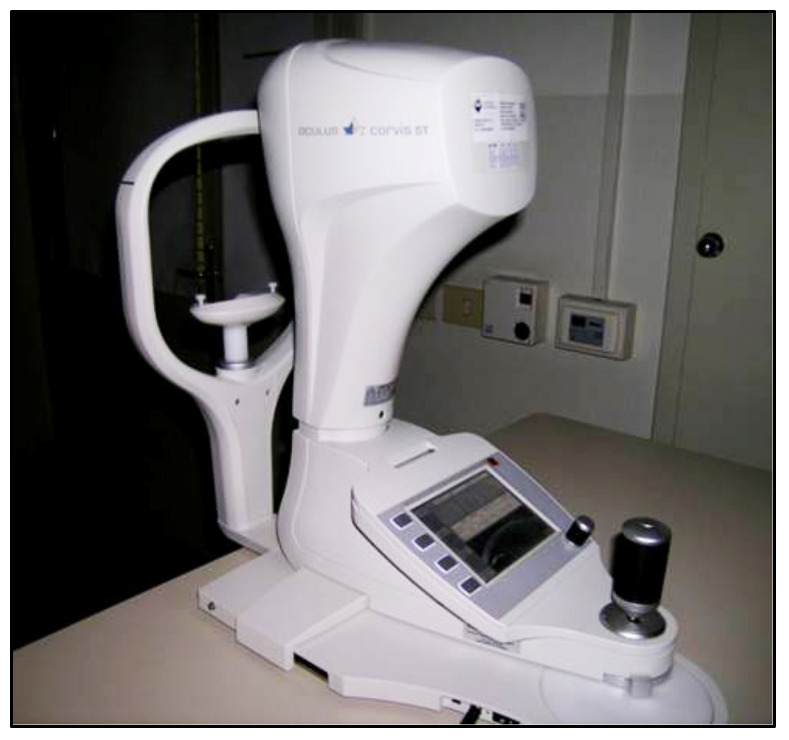
Corvis tonometer.

The Corvis ST (Oculus, Wezlar, Germany), a novel non-contact instrument, released in 2011 [61], is based on the system that causes indentation of the cornea by a jet of air. The tonometer has a built-in Scheimpflug ultra-high-speed device (Figure 8).

This instrument provides IOP measurements based on the indentation principle, in addition to pachymetry taken by on optical device and other biomechanical parameters of the cornea obtained by registering the surface deformation due to an applied air pulse, similar to an ORA device. A Scheimpflug camera visualizes an 8.5 mm diameter of the center of the surface of the cornea and precisely records the corneal deformation induced by the air-jet and its return to its normal shape with a high resolution and more than 4300 frames per second. A biomechanically corrected IOP value (bIOP), which takes the individual corneal deformation parameters into account, is also provided by the device.

The Corvis ST precision for the CCT and IOP values has been shown to be excellent; however, it is moderate for the corneal deformation parameters [62,63,64]. Previous studies demonstrated that Corvis ST tends to underestimate IOP readings obtained with GAT [61,62,65,66]. The Corvis ST biomechanically corrected IOP values (bIOP) have been shown to be less influenced by the CCT and corneal biomechanics and to be more effective in measuring the IOP in subjects who underwent refractive surgery [67]. Moreover, the Corvis ST corneal deformation parameters have been shown to be effective in discriminating between normal and keratoconic eyes [68].

## 5. Pneumotonometry

**Figure 9:**
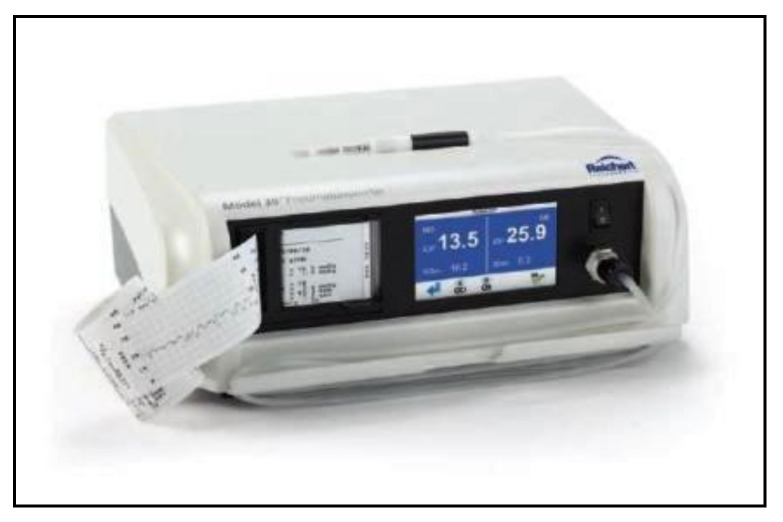
Pneumotonometer.

Pneumotonometers are devices based on the applanation principle, which use a different technology [69,70]: the tonometer probe consists of a hollow central tube flanked by a side exhaust, and the sensor is air pressure, which is dependent on the resistance of the exhaust. During the cornea applanation, the pressure within the central tubes increases to match the force generated by the IOP. A pneumatic electronic transducer converts the air pressure to a tracer on a strip of paper (Figure 9). 

**Figure 10:**
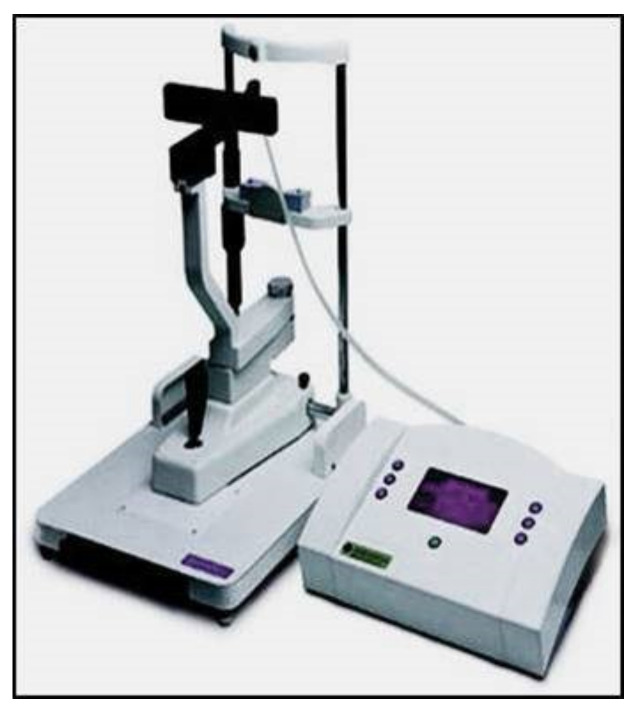
Langham Ocular Blood Flow pneumotonometer.

In several studies, pneumotonometry proved to be quite accurate and reliable in glaucoma screening and showed a greater reliability compared to GAT after PRK and LASIK [71,72,73]. Pneumotonometers such as the Pulsatile Ocular Blood Flow (OBF, Figure 10) have been used in the past to measure the pulse fluctuation and thereby give indirect information regarding the ocular blood pulse [74,75,76,77]. OBF measurements, however, appear to be more influenced by CCT and more variable than GAT readings, with a significant overestimation [78,79,80]. The clinical usefulness of this instrument in clinics still remains controversial.

## 6. Rebound Tonometry

**Figure 11:**
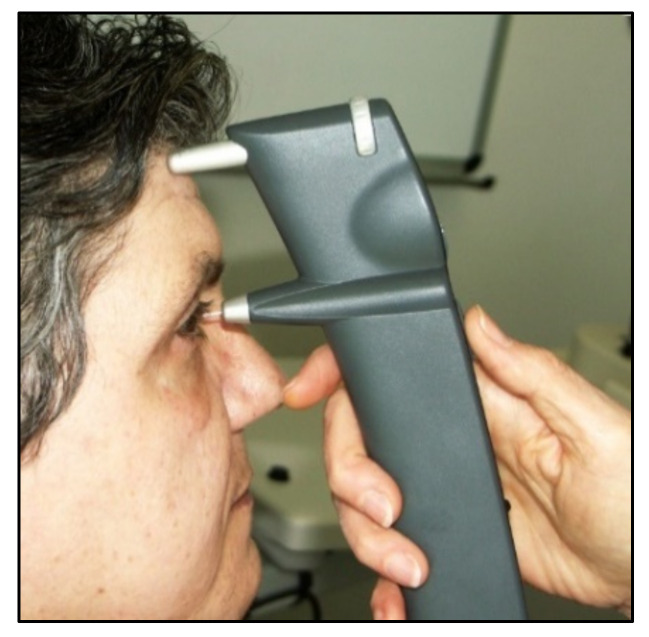
iCare rebound tonometer.

From a clinical point of view, the iCare rebound tonometer, introduced in 2000 by Kontiola [81], is currently one of the most interesting and widespread instruments used in practice (Figure 11).

**Figure 12:**
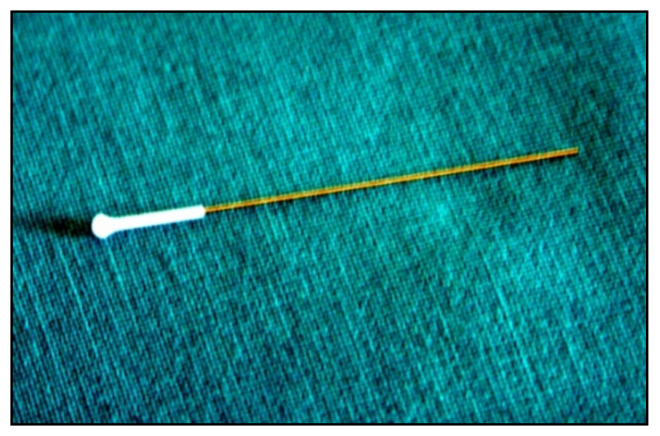
Disposable iCare probe.

A subtle probe impacts onto the cornea and then rebounds from the eye with a different velocity, which varies according to the IOP (Figure 12).

**Figure 13:**
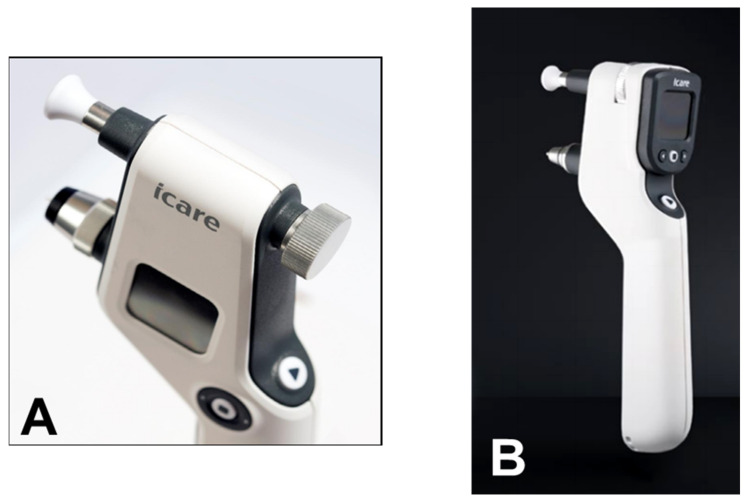
(**A**) iCare 100; (**B**) iCare 200 version.

The movement of the probe causes a voltage in the internal solenoid that is then amplified and digitally changed by a microprocessor. The IOP value is averaged from six consecutive measurements. The reliability of the final value is also displayed. The iCare tonometer is a reliable and precise instrument. It is rapid and easy to use, which is particularly helpful in busy clinics and with children, considering that there is no need for topic anesthesia [82,83,84,85]. The small surface contact makes it suitable to measure IOP after keratoplasty and in damaged corneas [85,86]. The iCare PRO version released in 2011 uses a shorter probe, which can also be used to measure IOP in a supine position. The most recent versions of this instrument, which are updated versions of the iCare PRO with a long probe (iCare IC100 and IC200) (Figure 13A,B), provide new features, such as a red or green light to show if the position of the probe is correct, in addition to providing the possibility of measuring IOP in a supine position [87,88].

**Figure 14:**
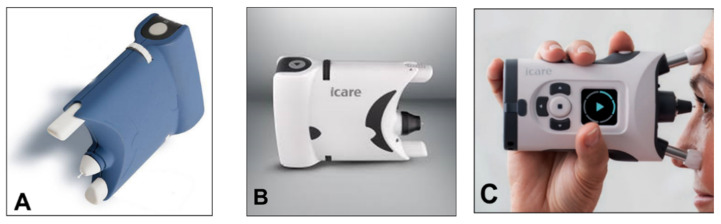
(**A**) iCare One; (**B**) iCare Home; (**C**) iCare Home2.

A simplified version (iCare One, replaced at first by the iCare Home and, recently, by the iCare Home2 (Figure 14A–C)), which can autonomously be used by patients, has recently been introduced for at-home auto-tonometry. It can be helpful for detecting IOP peaks, especially in suspect glaucoma and in normal tension glaucoma subjects, when IOP measurements appear to be normal during office hours [89,90,91,92].

Numerous studies have compared the different versions of iCare with GAT and other non-conventional tonometers. When compared to gold standard GAT, clinical results report a good correlation of tonometry readings, with r values greater than 0.8 for low-to-moderate GAT readings [93]. A recent study showed agreement between GAT readings and iCare to be good, with a <2 mmHg mean difference for all ranges of IOP [87]. For IOP > 23 mmHg, rebound tonometry tends to underestimate IOP compared to GAT, showing readings that are significantly lower [93].

Considering that rebound tonometry may be less traumatic on the cornea compared to GAT, it could offer a better alternative in post-operative patients to provide information regarding IOP. It is important to note that GAT measurements tend to be lower than iCare for post-operative patients with corneal edema [84,94]. The agreement between iCare and GAT has been reported to be acceptable in lamellar keratoplasty subjects; yet, it has been reported to be poor for patients with penetrating keratoplasty [84]. Rebound tonometry surely cannot replace GAT. However, it may prove to be clinically useful in post-operative eyes with fragile anterior segments, or eyes with increased risk of infection, in which GAT is impractical or not indicated.

In comparing the reliability and reproducibility of IOP values with iCare tonometry, air tonometry and GAT, Valero et al. reported ICC > 0.85 and low differences with GAT [95]. Several studies have reported that iCare provides reproducible and reliability measurements when compared to other tonometers, although it appears to slightly underestimate GAT readings [88,95,96,97,98,99,100,101]. The repeatability of iCare has been shown to be excellent, with ICC > 0.9 [88,99]. Reliability results with iCare compared to GAT have been shown to be good (ICC > 0.87) for patients with IOP with low-to-moderate measurements; however, such results are moderate (ICC = 0.52) for IOP < 16 mmHg and >23 mmHg [88]. Studies have reported sensitivity and specificity rates greater than 0.90 for rebound tonometry [98,99].

IOP readings taken with iCare do not appear to be affected by axial length, refractive error, age and gender [87,100]. GAT and rebound tonometry, however, are influenced by corneal characteristics. IOP tends to be overestimated for central corneal thicknesses greater than 520 microns [82,93,96,99,100,101]. Rebound tonometry also appears to be affected by corneal curvature, corneal hysteresis and disease [96,100,101]. Tonometer measurements tend to be more accurate with iCare for middle levels of IOP, ranging 16–23 mmHg [100].

Rebound tonometry has several advantages, which include having a short learning curve, being user friendly in nature, being well tolerated, and being safe for staff and patients. The instruments are portable, self-calibrated, affordable and do not require a slit lamp, topical anesthesia or fluorescein dye. These iCare instruments can also be used by trained non-medical staff. Multiple readings can be taken if needed without the fear of corneal abrasion or other complications [88]. Unlike GAT that uses a prism in contact with the cornea, the minimal contact and duration with the disposable iCare tips limits the risk of iatrogenic damage and cross-infection, which is of great importance in the recent COVID era [101]. Moreover, iCare does not induce IOP reduction caused by ocular massaging that can be observed in GAT [102]. The iCare home version can be useful in self-twenty-four-hour measurements of IOP to monitor diurnal variations in IOP, which may assist in treatment decision making and surgical timing, especially in patients at greater risks of IOP spikes such as pigment dispersion glaucoma, angle-closure suspects and pseudoexfoliation glaucoma [100].

The rebound tonometers offer numerous advantages; however, several cons should be noted. A recent study comparing iCare with GAT in 1000 eyes showed larger mean differences between tonometers in eyes with IOP > 22 mmHg and in the group of glaucoma patients with medications [103]. The precision and accuracy of iCare can be influenced by peripheral measurements of the cornea as opposed to proper central positioning of the tip [104]. Based on the good agreement with GAT for IOP values <21 mmHg, iCare can be a time-saving and wise alternative in a routine busy clinical screening setting, in which the majority of healthy patients show low-to-moderate IOP. These instruments can offer additional helpful information in a community-based setting when used together with other pertinent screening tools. High IOP readings taken with iCare tonometers need to be checked and confirmed with gold standard GAT. Rebound tonometry applies minimal pressure on the cornea; thus, it can be used to provide indicative IOP readings in first-day post-operative patients, keeping in mind the good, yet limiting, agreement with GAT readings. The ease of use, portability, rapidity, and use in supine positions make it an excellent tool for examining children, disabled and/or bedridden patients and patients with limited collaboration, in which GAT cannot be performed or is impractical.

## 7. Dynamic Contour Tonometry

**Figure 15:**
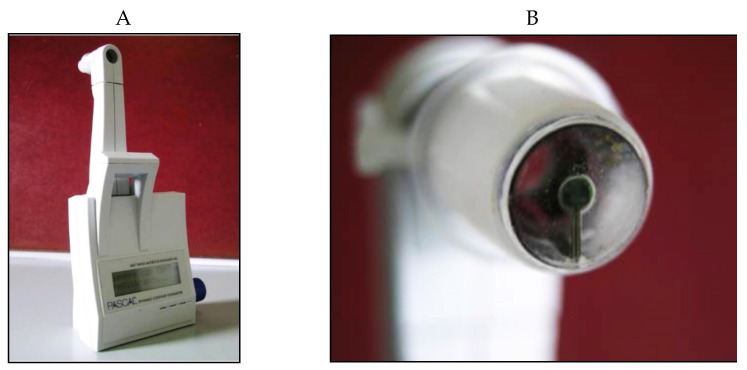
Dynamic Contour Pascal (**A**) with its sensor tip (**B**).

The Dynamic Contour Tonometer (PASCAL, DCT) (SMT Swiss Microtechnology AG, Port, Switzerland) is a relatively new device developed by Kaufmann et al. in 2003 [105] and implemented by Kanngiesser et al. in 2005 [106]. The DCT, which is not based on the applanation principle, calculates the IOP using the Pascal principle, according to which the pressure change is applied to all parts of a fluid in a contained enclosed space. The tonometer is positioned on the slit-lamp, requires the use of anesthetic drops (no fluorescein) and is automatically calibrated. It uses a concave contour tip that is equipped with a tiny sensor in the center of the contact surface (Figure 15).

When the “contour matching” between the surface of the cornea and the tip of the instrument is reached, the tangential forces of the cornea are cancelled, and the embedded pressure sensor directly measures the IOP without any cornea deformation and bias related to corneal factors, at least theoretically [107,108,109,110]. The pressure sensor tip is protected with a thin silicone membrane covered by disposable sensor caps in order to avoid the risk of infection. The DCT requires about 8-10 s of corneal contact in order to provide IOP measurements, which are shown on an LCD screen. Quality scores and ocular pulse amplitude (OPA) values are also provided. The DCT IOP is based on the diastolic IOP, which should be considered when comparing DCT and GAT IOP measurements. The OPA provides indirect information regarding perfusion of the choroid, which has been demonstrated to be important in the onset and progression of glaucoma [111]. 

The DCT has been shown to be less influenced by the properties of the cornea and can be therefore helpful in taking IOP readings in subjects with previous photorefractive surgeries [112,113,114,115,116]. The DCT has shown high precision, with higher reproducibility than GAT [109,117]. In comparison with GAT, however, the DCT IOP measurements, although highly correlated, tend to be significantly higher [26]. 

The principal drawbacks of the DCT include: the need for a slit lamp, anesthetic and corneal contact; the need for trained staff and highly cooperative patients that can keep a good head and eye position for at least 8 s, meaning that this tonometer may prove to be difficult to use and not rapid in busy clinics [118]; and reduced accuracy in the presence of irregular corneas [119].

## 8. Applanation Resonance Tonometry

**Figure 16:**
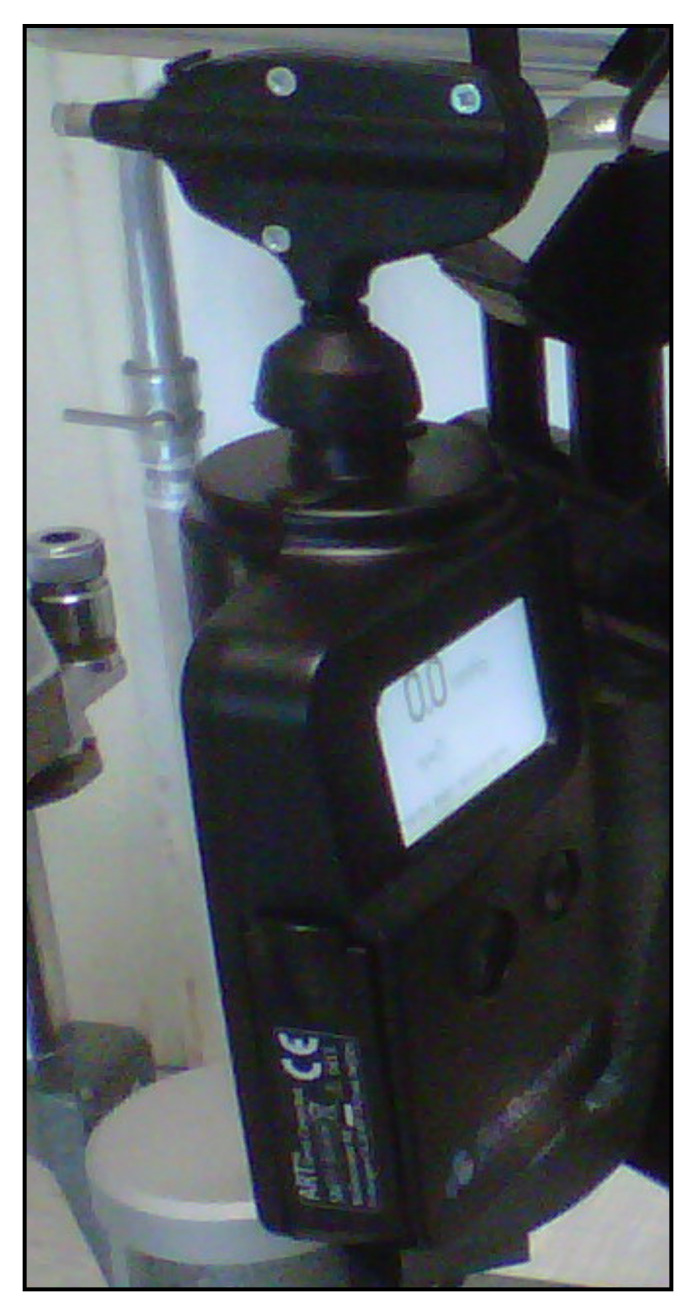
The BioResonator ART tonometer.

The Applanation Resonance Tonometer (ART), known in the current commercial version as BioResonator ART (BioResonator AB, Umea, Sweden) (Figure 16), was developed by Eklund et al. in 2003 [120]. It was released as both a manual and automatic version in 2012 [121]. This tonometer uses the applanation tonometry principle combined with the resonance technique. The device needs must be mounted on a slit lamp, requires the use of local anesthetic drops before IOP measurement and uses a concave surface sensor tip, which is positioned on the cornea. The sensor tip is manually pushed towards the cornea in the manual version of the instrument, whereas the automatic version provides a tiny motor for movement of the tip. A resonance piezoelectric device is found in the tip of the sensor that generates a shift in frequency which is proportional to the area of contact. The IOP is based on the contact area and force measurement parameters, which are taken continuously throughout the test [120,121].

The ART probe must be carefully disinfected before each subject. This tonometer is self-calibrated and gives the repeated IOP measurement median and a quality index reflecting the standard deviation of the IOP values. The IOP measurement provided by the BioResonator ART is claimed to be more accurate than that of GAT considering that it represents the median of repeated measures; however, the precision of the instrument has been questioned [122,123,124].

Previous studies have reported that this tonometer can provide an overestimate in IOP values when compared to GAT [123,124,125]. Furthermore, it seems to be affected by CCT and corneal biomechanics [120,123,126]. Despite some advantages (repeatable and reliable measurements, no fluorescein is needed), the BioResonator ART has some drawbacks that may reduce its clinical usefulness, including: the need for a slit lamp, anesthetic and corneal contact, with sterilization issues; can be affected by various artifacts and measurement errors; moreover, its accuracy is influenced by the thickness and biomechanics of the cornea.

## 9. Continuous IOP Monitoring

All the aforementioned devices can be usefully employed for taking spot IOP measurements during office time. This can be acceptable in a screening setting, but, unfortunately, undetected elevated IOP spikes tend to occur during the night in many glaucomatous patients [127]. IOP readings during clinical office hours fail to detect these peaks in more than 50% of cases with a significant underestimation of IOP [128,129,130,131]. Hughes et al. reported that data obtained with continuous monitoring in IOP using a 24 h device had an influence in therapeutic decisions in 79.3% of enrolled subjects [132]. Keeping these data in mind, it can be inferred that our current standards in clinics with regard to taking IOP measurements may not suffice and thus need to be modified [133].

An important step towards a more precise management of patients affected by ocular hypertension and chronic glaucoma would be the possibility of continuously monitoring IOP values not only during the day but also in the night, as occurs with the 24 h blood pressure Holter. This information could be particularly useful in the so-called normal tension glaucoma patients, which show significant damage progression despite an apparently normalized IOP. In these cases, an elevated IOP can sometimes be found during the night, especially early in the morning, outside office hours [134,135]. A number of devices, most of them only experimental, have been proposed for this purpose over the past 20 years [136,137,138]. Some of them need to be surgically inserted into the eye, either during a cataract extraction procedure, usually embedded in an intraocular lens [139,140,141], or positioned in the anterior chamber [142,143], or in the suprachoroidal space [144]. A non-invasive continuous IOP measurement is also possible using special contact lenses with different types of miniaturized sensors and a wireless power transmission of data to a recorder.

**Figure 17:**
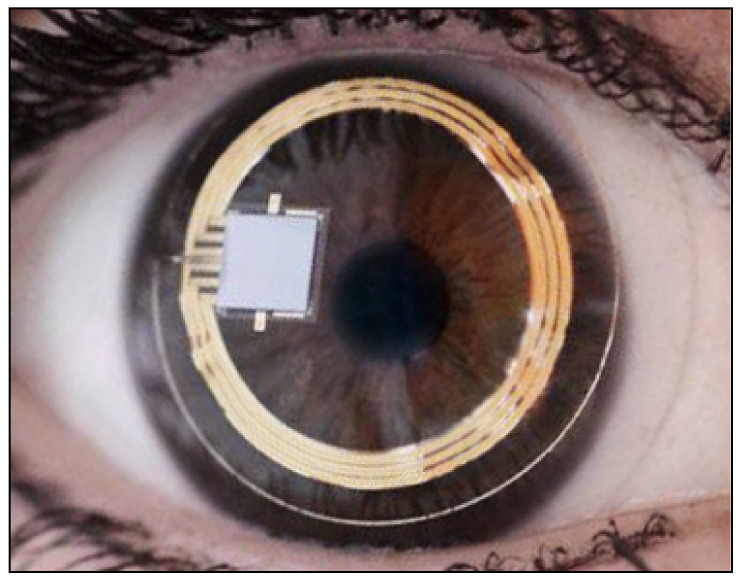
Sensimed Triggerfish.

**Figure 18:**
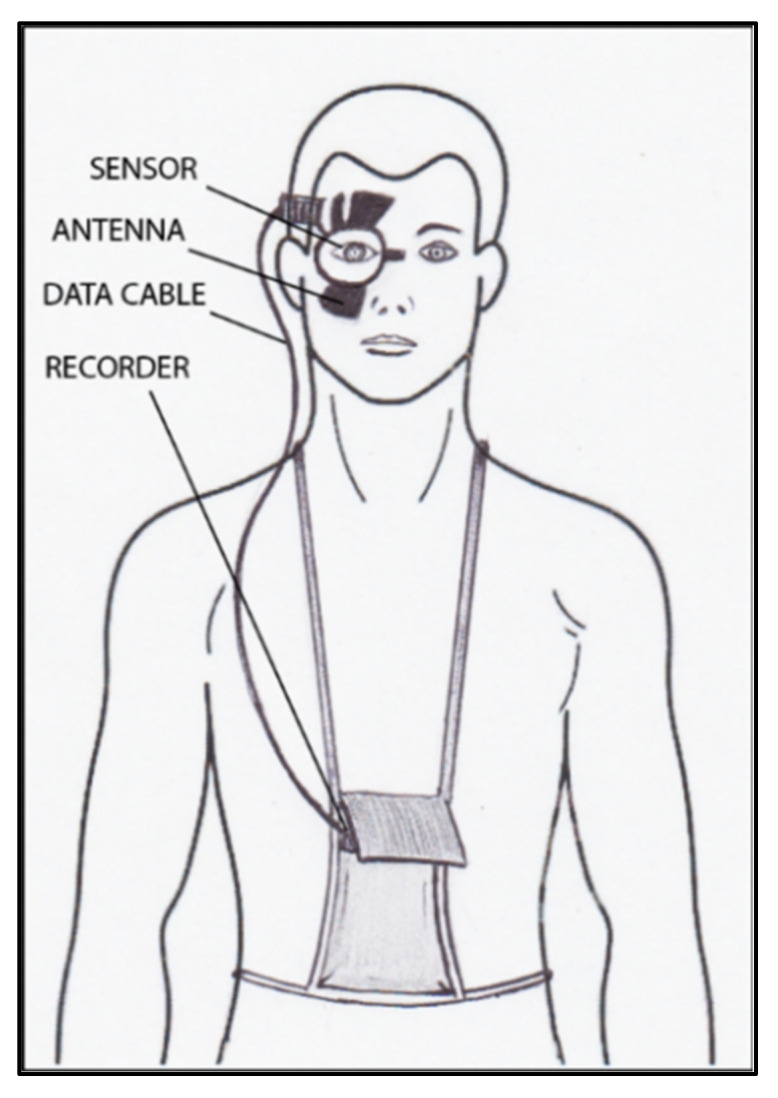
Schematic view of Triggerfish, wireless antenna and portable recorder.

The contact lens sensor Sensimed Triggerfish (Triggerfish CLS, Sensimed AG, Lausanne, Switzerland) is a miniaturized electromechanical system with a microprocessor embedded in a disposable silicon contact lens, which transmits a signal to an external wireless antenna located in the periocular surface (Figure 17 and Figure 18). The data are then transferred to a portable recorder, for a total of 288 data sets in 24 h. This device can measure small modifications in the curvature of the cornea believed to be due to variations of IOP [145,146,147,148,149].

Triggerfish CLS is usually well tolerated [150,151,152] and has also been shown to have high reproducibility [150,151,152,153,154,155]. The information obtained with these device parameters might be useful in assessing changes and IOP fluctuations in subjects with pseudoexfoliation syndrome, pigment dispersion, and in predicting the visual field loss progression rate [156,157]. Several studies have shown the usefulness of this contact lens sensor for assessment of the risk of glaucoma, which may prove to be important in subjects with NTG, in which IOP tends to be normal with diurnal readings [158,159,160,161].

The main problem with this device is that there is no direct correlation between corneal changes, expressed in millivolt equivalent (mVeq), and IOP values. Studies have shown that IOP measurements taken with GAT and Triggerfish values tend to have a high correlation at the beginning, after the insertion of CLS [148]; however, the correlation becomes poor after 24 h [153,154].

CLS is advantageous because it is not invasive, can be easily removed and dismantled [155], readily available [155], accepted and tolerated by patients [150,151,152], and provides good reproducibility [151,153,154]. The validity (i.e., considering the estimation accuracy of IOP readings) and relatively costly equipment of CLS are important drawbacks, which render the clinical usefulness of this instrument still debatable in literature [153,154].

Other types of devices able to measure IOP, either implantable or non-invasive [162,163,164,165,166,167,168,169,170,171,172,173], have been proposed, but almost all are still experimental and need further studies before being introduced into clinical practice.

## 10. Conclusions

As shown in Table 1, numerous tonometers have been proposed in the past. It is important to note that in managing and treating glaucoma patients, it is preferable and more reliable to measure IOP every time with the same type of equipment for each individual glaucoma patient. Several instruments have provided specific advantages compared to Goldmann tonometry. Alternative systems reported in literature, either non-invasive or implantable, remain experimental. Despite promising preliminary results, none have obtained widespread use and are adaptable in a routine clinal setting. New is not always better. The Goldmann tonometer, despite its limitations and a lack of innovative and novel advancements in the past 70 years, continues to be theoretically more precise and considered the gold standard tonometer to diagnose and manage patients with ocular hypertension and glaucoma.

## Figures and Tables

**Table 1:** Summary of the characteristics of the various available tonometers.

Tonometer Type	Production Year	Working Principle	Contact/ Noncontact	Advantages	Disadvantages	Clinical Suitability	Cost *
Schioetz tonometer	1905	indentation	contact	Simple	High variability	still used only in developing countries	feasible
				Inexpensive	Can be used in a supine position only		
				No need of slit-lam, electricity or charging batteries	Affected by various sources of error		
					Topical anesthesia is needed		
Goldmann applanation tonometer (GAT)	1955	applanation	contact	Quite simple to use	Affected by corneal thickness and other corneal parameters	good	feasible
				Accurate and reproducible measurements	The accuracy depends on the clinician’s experience		
				It is currently considered as the gold standard in IOP measurement	Needs to be used with a slit-lamp		
					Can be used in the upright position only		
					Topical anesthesia and fluorescein are needed		
Perkins tonometer	1965	applanation	contact	Can be used in a supine position too	Affected by corneal thickness and other corneal parameters	good	feasible
					Topical anesthesia and fluorescein are needed		
TonoPen	1989	indentation/ applanation	contact	Lightweight and portable	Hight variability and quite poor repeatability	moderate	feasible
				Quick and simple to use	Can underestimate IOP values		
				Can be used in any position	Topical anesthesia is needed		
				No need of slit-lamp or electricity	Influenced by corneal parameters		
				Self-calibration, provides quality index			
Air-puff tonometers	1973	applanation	noncontact	Easy and fast to use	Need of regular calibration	ideal as a screening tool	medium
				No need to touch the cornea	Readings are device-dependent		
				No need of topical anesthesia and fluorescein	Possible germs aerosol		
				Can be used by paramedical staff	Influenced by corneal parameters		
					Less accurate when IOP > 20 mmHg		
Ocular Response Analyzer	2005	applanation	noncontact	Simple to use, self-calibration, provides quality index	Possible germs aerosol	good	expensive
				No need of topical anesthesia and fluorescein			
				Can be used by paramedical staff			
				Provides additional information (corneal central thickness and biomechanics)			
				IOP correction for corneal biomechanical parameters			
				Useful after corneal refractive surgery			
				Detection of corneal diseases			
Corvis ST	2011	indentation/ applanation	noncontact	Simple to use, self-calibration, provides quality index	Tends to underestimate the GAT IOP values	good	expensive
				No need of fluorescein and topical anesthesia			
				Can be used by paramedical staff			
				Provides additional information (corneal central thickness and biomechanics)			
				IOP correction for corneal biomechanical parameters			
				Useful after corneal refractive surgery			
				Detection of corneal diseases			
Pneumotonometers	1969	applanation	contact	OBF provides information on the ocular blood pulse	Affected by corneal thickness	controversial	expensive
					Overestimates IOP values		
iCare tonometer	1997	ballistic probe (rebound)	contact	Ease of use with a short learning curve	Needs a proper central positioning of the tip	excellent	feasible
				Portable and self-calibrated	Influenced by corneal thickness		
				No slit-lamp or topical anesthesia or fluorescein dye required			
				Can be used by trained non-medical staff			
				Can be used in supine positions (iCare PRO and iCare 200)			
				Minimal corneal trauma (useful in post-operative patients)			
				The home version can be useful in self-twenty-four-hour monitoring of IOP			
Dynamic contour tonometer (DCT, PASCAL)	2005	contour matching	contact	No need of fluorescein, disposable probes	Need of slit lamp and topical anesthesia	poor	medium
				Self-calibration, provides quality index	Difficult to use		
				Independent from corneal properties	Need of highly cooperative patients		
				Useful after corneal refractive surgery			
				High precision			
				Additional information (ocular pulse amplitude)			
BioResonator ART	2003	applanation	contact	No need of fluorescein	Need of slitlamp and local anesthetic	moderate	medium
				Self-calibration, provides quality index	Need of probe disinfection		
				High reliability (median of repeated IOP measurements)	Required training to use		
					Affected by corneal properties		
Sensimed Triggerfish	2004	corneal curvature monitoring	contact	Continuous measurements over a 24-hour period	It does not provide direct IOP values	moderate	feasible
				Good tolerability	IOP estimation accuracy not known		
				High reproducibility			

* feasible: 0–5000 euros; medium range 5000–10,000 euros; expensive: >10,000 euros.
